# Evaluation of Novel GLP‐1 analogue in the treatment of Alzheimer’s disease

**DOI:** 10.1002/alz.089799

**Published:** 2025-01-09

**Authors:** Paul Edison

**Affiliations:** ^1^ Imperial College London, London, United Kingdom; Division of Neurology, Department of Brain Sciences, Imperial College London, United Kingdom, London, London United Kingdom

## Abstract

**Background:**

Liraglutide is a glucagon‐like peptide‐1 (GLP‐1) analogue licensed for the treatment of type 2 diabetes mellitus (T2DM). Preclinical evidence in transgenic models of Alzheimer’s disease suggests that liraglutide exerts neuroprotective effects by reducing amyloid oligomers, normalising synaptic plasticity and cerebral glucose uptake, and increasing the proliferation of neuronal progenitor cells.

**Method:**

This is a multi‐centre, randomised, double‐blind, placebo‐controlled, phase IIb trial of liraglutide in participants with mild to moderate Alzheimer’s dementia, conducted at several centres in the UK.

As a part of this study, MRI brain scans of all patients were performed at baseline and after 12 months treatment with liraglutide or matching placebo along with neuropsychometric evaluation and [18F]FDG PET. A total of 204 Alzheimer’s participants were randomised to receive either liraglutide or placebo as a daily subcutaneous injection for 12 months. All subjects had regular clinical visits and neuropsychometric evaluation at regular intervals. Repeat scans were performed in all subjects who completed 52 weeks of treatment. Volumetric changes from baseline to follow‐up in MRI scans were evaluated using both regional volume analysis and voxel‐based morphometric analysis.

**Result:**

MRI analysis demonstrated a slower decline of temporal lobe volume and total grey matter volume in liraglutide‐treated patients compared to the placebo group. Voxel‐based morphometry (VBM) analysis demonstrated that liraglutide‐treated participants showed a slower reduction in whole cortical grey matter, frontal, temporal and parietal lobe volume in participants treated with liraglutide compared to placebo. This was associated with slower decline in cognitive function.

**Conclusion:**

These findings highlight the potential of GLP‐1 analogues in the treatment of Alzheimer’s disease

Authors:

**Edison P,** Femminella GD, Ritchie C, Holmes C, Nowell J, Walker Z, Ridha B., Raza S, Livingston NR, Frangou E, Love S, Williams G, Lawrence R, Mcfarlane B, Archer H, Coulthard E, Underwood B. R, Koranteng P, Karim S, Bannister C, Perneczky R, Prasanna A, Junaid K, McGuinness B, Nilforooshan R, Macharouthu A, Donaldson A, Thacker S, Russell G, Malik N, Mate V, Knight L, Kshemendran S, Holscher C, Mansouri A, Mae Chester‐Jones L, Holmes J, Tan T, Williams S, Brooks DJ, Harrison J, Hinz R, Tadros G, Passmore AP, Ballard C.

